# Epidemiological and clinical characteristics of 66 Tunisian Sickle cell syndrome patients

**DOI:** 10.4314/ahs.v23i3.26

**Published:** 2023-09

**Authors:** Ahlem Sahli, Faida Ouali, Rym Dabboubi, Sondess Hadj Fredj, Nabila Meddeb, Naila Mzoughi, Taieb Messaoud

**Affiliations:** Laboratory of Biochemistry and Molecular Biology Children's Hospital Bechir Hamza (LR00SP03)

**Keywords:** Acute complications, chronic complications, sickle cell syndrome, steady state

## Abstract

**Introduction:**

Sickle cell syndrome (SCS) represent a real health problem. In this work, we propose to study the epidemiological and clinical features of 66 patients with SCS.

**Methods:**

This is a retrospective descriptive cross-sectional study carried out on a population of 66 patients with SCS, (36 S/S, 18 S/β-thalassemia, seven S/C and five S/O_Arab_), over a period of two years.

**Results:**

The average age of our population is 15.5 years ± 8.4. 36 patients (55%) were born to a consanguineous marriage and 35 (53%) had siblings with SCS. The average baseline hemoglobin in our patients is 9.1g/dL±1.51. S/C patients have significantly higher baseline hemoglobin than S/S, S/β-thalassemia and S/OArab with p <0.05. Jaundice, mucosal skin pallor and hepatomegaly have been observed only in S/S, S/β-thalassemia and S/_OArab_ patients. The persistence of splenomegaly is more frequent in S/C than in S/S, and in S/-thalassemia than in S/S. The most common acute complications were vaso-occlusive attacks (69.7%) and worsening of anemia (54.54%). The most common chronic complication was cholelithiasis (36.36%).

**Conclusion:**

S/C patients present the best tolerated form and were the least affected by chronic complications and therefore can lead an almost normal life.

## Introduction

Sickle cell disease (SCD) is an autosomal recessive genetic disorder that affects the red blood cells 1. It is the most common genetic disease in the world and it represent a real health problem due to its frequency and chronic evolution 2.

The pathophysiology of SCD is influenced by various genetic and environmental factors that come up with highly variable clinical expression including inflammation, hemolysis, micro- and macrovascular obstruction and organ damage. The management of SCD is multidisciplinary and requires standardization of its complications or phenotypes 1.

Sickle cell syndrome (SCS) include: homozygous sickle cell disease β^S^/β^S^ and double heterozygous β^S^/β^X^; this is the heterozygous association of hemoglobin S with a lesion of the other gene β (β-thalassemia or other abnormal hemoglobins such as hemoglobin C, hemoglobin O_Arab_).

In Tunisia, the most frequently encountered hemoglobin abnormalities are β-thalassemia with a prevalence of 2.21% and sickle cell disease with a prevalence of 1.89%. While Hb C and Hb OArab are more rare, their prevalence is about 0.37%. The north-western Tunisia, is the most concerned 3.

Hemoglobin S (HbS) results from a mutation in the 6th codon of the β gene located on chromosome 11. It is due to the replacement of glutamic acid by valine. Hemoglobin C results from a point mutation leading to the replacement of glutamic acid by lysine at position 6 while hemoglobin O_Arab_ results from the substitution of glutamic acid by lysine at position 121 on the same chromosome. As for the β-thalassemia trait, 31 mutations have been identified in Tunisia. The most frequent are codon 39 (C>T) and IVS I-110 (G>A) 2.

In SCS, the heterozygous association of HbS with a lesion of other β gene can affect the clinical presentation of the disease. So, a great heterogeneity in SCS clinical expressions was described with only 15% of patients developing severe disease 4.

In order to show this disparity in the clinical expression, we propose to study the epidemiological and clinical particularities of 66 patients with SCS.

## Methods

This is a descriptive retrospective study of SCS patients' records over a two-year period.

Our study population consists of 66 patients with SCS (36 homozygous S/S sickle cell patients, 18 S/β-thalassemia patients composed by S/β^o^ thalassemia and S/β^+^ thalassemia phenotypes, 7 S/C patients, and 5 S/O_Arab_ patients).

**Inclusion criteria:** Carriers of sickle cell syndrome, diagnosed during an anemia exploration or during a hospitalization for an inaugural complication of the disease, or during family investigations carried out systematically in the siblings of patients already being followed or after neonatal screening. These patients are regularly monitored during quarterly visits to the outpatient Hemoglobinopathy clinic at the Children's Hospital in Tunis.

### Epidemiological data

Current age, age of diagnosis, origins, siblings and consanguinity were collected from patients' medical records.

### Clinical data

Three types of situations have been described: a steady state defined by the absence of any fever, vaso-occlusive or hemolytic episode, acute complications and chronic complications phases.

Blood counts were performed by flow cytometry on the Beckman LH750TM Hematology analyzer (Bechman Miami, FL, USA). Hemoglobin was determined by high performance cation exchange liquid chromatography (HPLC) using the Variant II hemoglobin analyzer (Biored Laboratories, Hercules, CA, USA).

The complications and the level of Hb F reported in this work were noted before the intake of hydroxyurea by some patients.

### Statistical Analysis

The data collected was processed using SPSS version v20.0 (Statical Package for Social Sciences). Results for the quantitative variables are expressed in terms of means ± standard deviation. If the standard deviation is greater than the mean, the results are expressed in terms of the median [minimum-maximum]. The STUDENT t-test was used to compare the means of the quantitative variables. Inter-group comparisons for qualitative variables were performed by the « chi2 » test. Comparison of the means of different variables with small numbers (n<10) was tested by the analysis of variance (Mann Whitney). The significance threshold for the statistical tests was set at p < 0.05.

## Results

Our population is composed of 66 patients. The average age is 15.5 ± 8.4 years with age interval ranging from one year to 49 years. In this population, 36 patients (55%) were born to a consanguinous marriage and 35 (53%) had siblings with SCS.

**Figure 1:**
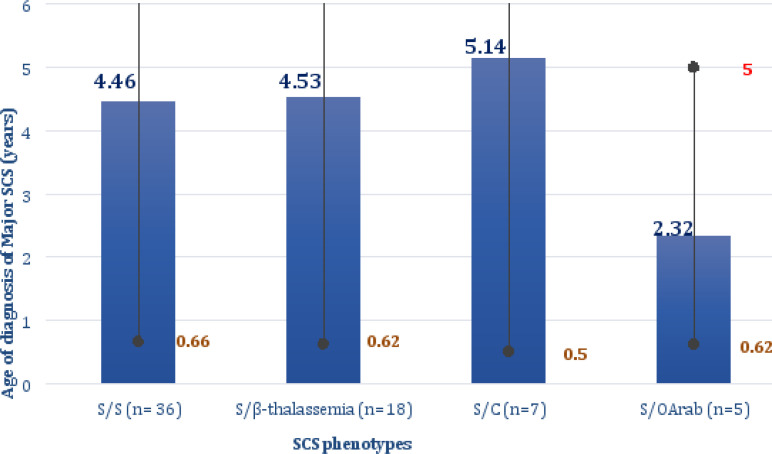
Age of diagnosis of SCS patients by phenotype In blue: Average age of diagnosis, In green: Minimum age of diagnosis, In red: Maximum age of diagnosis, n: Number of patients

The mean age at diagnosis is 3.8 ± 2.6 years. 33 of our patients (50%) were diagnosed before the age of two years. Among them, 24 patients (36.36%) (14 S/S, seven S/β-thalassemia, one S/C and two S/O_Arab_) were diagnosed before the age of one year. The mean age of diagnosis of SCS patients by phenotype is shown in (Figure 1).

In the Steady state, the mean baseline hemoglobin in our patients is 9.1±1.51 g/dL, it is 8±1.1 g/dL in S/S, 8.2±1.2 g/dL in S/β-thalassemia, 11± 1.4 g/dL in S/C and 9.4± 1.2 g/dL in S/O_Arab_.

S/C patients have significantly higher baseline hemoglobin than S/S, S/β-thalassemia and S/OArab with p<0.05. For each patient, hemoglobin fractions were studied at the time of diagnosis.

The rate of HbF in patients whose age at diagnosis ≤ five years is 21.7± 15.61% while the rate of HbF in patients whose age at diagnosis >5 years is 14.28 ± 9.68% (p=0.0928).

Jaundice and Pallor was observed respectively in 24.24% and 36.36% patients.

19 patients (28.7%) had a splenomegaly at the time of the last medical visit ranging from a spleen tip (<2cm) to 17cm from the left costal margin. They are divided into six S/S patients (16.6%), nine S/β-thalassemia patients (50%) and four S/C patients (57.1%). Their average age is 15.83 ± 8.9 years. The persistence of splenomegaly beyond the age of five years is significantly more frequent in S/C than in S/S (p=0.02), and in S/β-thalassemia than in S/S (p=0.009).

Ten patients (15.1%) had splenic atrophy at the time of data collection; including eight S/S and two S/O_Arab_. The remaining patients either had a normal spleen volume or had undergone splenectomy.

14 patients (21.21%) had hepatomegaly, including 12 S/S, one S/β-thalassemia and one S/OArab. Their mean age is 17.53 ± 5.18 years.

SCS acute complications and their frequencies are reported in (Table 1).

**Table 1:** Acute complications

Complications		SCS *(n=66)*	*S/S(n=36)*	S/β-thalassémia *(n=18)*	S/C*(n=7)*	S/O_Arab_ *(n=5)*	P
VOC*		**69.7%** (n=46)	**75%** (n=27)	**61.11%** (n=11)	**57.14%** (n=4)	**80%** (n=4)	P1: 0.29 P2: 0.33 P3: 0.85 P4: 0.4
**Acute worsening of anemia**		**54.54%** (n=36)	**55.55%** *(n=20)*	**66.66%** *(n=12)*	**28.57%** *(n=2)*	**40%** *(n=2)*	P1:0.443 P2: 0.24 P3: 0.17 P4 :1
**Infectious Complications**	*Total infections*	**44%** (n=29)	**47.22%** (n=17)	**33.33%** (n=6)	**14.28%** (n=1)	**100%** (n=5)	P1: 0.33 P2: 0.1 P3: 0.34 **P4:0.003**
	*Osteoarticuhr*	**9.09%** (n=6)	**13.88%** (n=5)	0	0	**20%** *(n=1)*	P1: 0.096 P2: 0.29 P3: NA P4: 0.26
	*pulmonary*	**22.72%** (n=15)	**25%** (n=9)	**11.11%** (n=2)	**14.28%** *(n=1)*	**60%** (n=3)	P1: 0.23 P2: 0.53 P3: 0.82 P4: 0.097
	*Urinary*	**12.12%** (n=8)	**8.33%** (n=3)	**22.22%** (n=4)	0	**20%** *(n=1)*	P1: 0.15 P2: 0.42 P3: 0.17 P4: 0.21
ATS*		**16.66%** (n=11)	**19.44%** (n=7)	0	0	**80%** (n=4)	**P1: 0.041** P2: 0.19 P3: NA **P4: 0.003**
ASS*		**9.09%** (n=6)	**8.33%** (n=3)	**16.66%** (n=3)	0	0	P1: 0.35 P2: 0.42 P3: 0.24 P4: NA
CVA*		**1.51%** (n=1)	**2.77%** (n=1)	0	0	0	P1: 0.47 P2: 0.65 P3: NA P4: NA
**Priapism**		**1.51%** *(n=1)*	**2.77%** *(n=1)*	0	0	0	P1: 0.47 P2: 0.65 P3: NA P4: NA

*ATS: *Acute thoracic syndrome*, *CVA cerebral vascular accidents, *VOC: *vaso-occlusive crisis*, ASS*: *Acute Splenic Sequestration* *NA *not applied*.

**P1 =** Significance rating of the comparison of means between the **S/S** and **S/β-thalassemia** groups

**P2 =** Significance rating of the comparison of means between **the S/S and S/C** groups

**P3 =** Significance rating of the comparison of means between the **S/β-thalassemia** and **S/C** groups

**P4:** Significance rating of the comparison of means between the **S/C and S/O_Arab_** groups

The frequency of vaso-occlusive crisis per year according to the phenotype of SCS patients is reported in (Table 2).

**Table 2:** Frequency of vaso-occlusive crisis per year according to the phenotype of SCS patients

Genotype frequencies	SCS *(n=66)*	S/S *(n=36)*	S/β-thalassémia *(n=18)*	S/O_Arab_ *(n=5)*	S/C *(n=7)*
**0**	**30.30%** *(n=20)*	**25%** *(n=9)*	***38.88%*** *(n=7)*	***20%*** *(n=1)*	***42.85%*** *(n=3)*
**1-2/years**	***30.30%*** *(n=20)*	***41.66%*** *(n=15)*	***22.22%*** *(n=4)*	**20%** *(n=1)*	***0*** *(n=0)*
**≥3/year**	***39.39°%*** *(n=26)*	***33.33%*** *(n=12)*	***38.88%*** *(n=7)*	***60%*** *(n=3)*	***57.14%*** *(n=4)*

SCS chronic complications and their frequencies are reported in (Table 3).

**Table 3:** Chronic complications in SCS patients

Complications	SCS *(n=66)*	S/S *(n=36)*	S/β-thalassémia *(n=18)*	S/C *(n=7)*	S/O_Arab_ *(n=5)*	P
**Gall bladder lithiasis**	**34.84%** (n=23)	**52.77%** *(n=19)*	**22.22%** *(n=4)*	0	0	P1: 0.24 P2: **0.014** P3: 0.079 P4: NA
Heart complications*	**16.66%** (n=11)	***13.88%*** *(n=5)*	**16.66%** *(n=3)*	**42.85%** *(n=3)*	0	P1: 0.78 P2: 0.071 P3: 0.16 P4: 0.09
**Aseptic osteonecrosis of femoral or humeral heads**	**6.06%** ***(n=4)***	**8.33%** *(n=3)*	**5.55%** *(n=1)*	0	0	P1: 0.71 P2: 0.42 P3: 0.52 P4: NA
**Leg skin ulcers**	**3%** ***(n=2)***	**5.55%** *(n=2)*	0	0	0	P1: 0.308 P2: 0.523 P3: NA P4: NA
**Sickle cell disease nephropathy**	**3%** (n=2)	**5.55%** *(n=2)*	0	0	0	P1: 0.3 P2: 0.52 P3: NA P4: NA
**Sickle cell disease retinopathy**	**1.51%** (n=1)	**2.77%** (n=1)	0	0	0	P1: 0.47 P2: 0.65 P3: NA P4: NA

***Heart complications*** *are limited to the auscultation of a heart murmur with a Doppler echocardiogram within the normal rang*e, * Sickle cell disease Nephropathy *was* diagnosed by the presence of 24-hour urine proteinuria, *NA: *not applied*.

**P1 =** Significance rating of the comparison of means between the **S/S** and **S/β-thalassemia** groups

**P2 =** Significance rating of the comparison of means between **the S/S and S/C** groups

**P3 =** Significance rating of the comparison of means between the **S/β-thalassemia** and **S/C** groups

**P4:** Significance rating of the comparison of means between the **S/C and S/O_Arab_** groups

## Discussion

The age of our patients ranges from one to 49 years with a median of 16 years. Patients over 18 years of age refuse to be transferred to adult hematology consultations and continue to be followed in our hospital by the same medical and paramedical staff with whom they have established a relationship based on trust and understanding, thus emphasizing the psychological component in the management of SCS. Consanguinity which increases the risk of sickle cell disease, is at about 55% in our cohort. It is at 32% in the Tunisian population and can reach up to 60% in rural areas 5.

The number of patients with affected siblings is at 53%. This is due either to an undiagnosed disease in the first affected child, or to a genetic counselling not provided or poorly assimilated during subsequent pregnancies leading to failure to perform prenatal diagnosis (PND), or to the refusal of therapeutic termination of pregnancy (TTI) when the PND identifies a sick foetus. PND in Tunisia is offered free of charge to at risk couple as part of prevention programme 6.

The average age of diagnosis for our patients was 3.8 years. This result is similar to that found in a study conducted in the Congo, which reported a mean age of diagnosis of 3.2 years 6. 50% of our patients were diagnosed before the age of two years. This is partly related to the severity of SCS in Tunisia, characterized by a Beninese haplotype in 95% of cases 3. Family surveys systematically performed for the siblings of patients already followed up and neonatal screening performed for 10 of our patients were also responsible for the early diagnosis of the disease. This is also the case of S/C patients diagnosed relatively early despite the fact that they present a moderate and well-tolerated form and would therefore be diagnosed late.

In the steady state, anemia is normocytic normochromic in S/S, S/O_Arab_ and S/C patients. In S/β-thalassemia patients the anemia is microcytic hypochromic. The microcytosis being explained by the presence of the β-thalassemic trait.

In our study, S/C hemoglobinopathy is distinguished from other SCS by moderate, or in some cases even absent, anemia (average baseline Hb level of 11 g/dL up to 13 g/dL). Their baseline hemoglobin level was found to be significantly higher than that of S/S, S/β-thalassemia and S/OArab. This has been confirmed in some studies reporting near-normal baseline hemoglobin levels in S/C patients 5, 7. These data are indicative of chronic hemolysis in the different phenotypes of SCS, which remains less intense in S/C patients, so that, anemia is milder 8-9. According to Ronald L Nagel et al, the ratio of HbS to HbC (50/50) explain moderate hemolysis in SC patient 9.

The Hight Hb F level noted in our patients does not agree with the fact that 95% of sickle cell patients in Tunisia have a Beninese haplotype 3 which is known for a low Hb F level of around 7% 10. So other genetic markers that have an implication on the Hb F expression rate should be looked for in our population. A co-inheritance of 3,7 Kb alpha-thalassemia at the heterozygous state was found in four patients SS of our population. The association with alpha-thalassemia plays a modulating role by reducing the risk of some sickle cell complications which vary between studies. In fact, there is lack of consensus on its impacts on the biological and clinical manifestations of SCD patients 11.

Jaundice was observed in 24.24% patients. In a similar study, Thiam found jaundice in 36.9% of cases. Shongo found it in 63.4% of cases 12. Jaundice due to severe hemolysis usually appears after the age of six months, when fetal hemoglobin begins to be replaced by hemoglobin S, which then becomes predominant 12.

Cutaneous mucosal pallor indicative of chronic anemia was found in 36.36% patients. In Moroccan research, Da Silva reported pallor in 51.5% 12, 13, while in another study in Burkina Faso, Solange reported pallor in 43.6% of patients 8.

Jaundice and cutaneous mucosal pallor have not been reported in S/C patients confirming moderate hemolysis.

Splenomegaly was noted at the last medical visit in 28.7% of our patients with an average age of 15.83 ± 8.9 years. It affected 16.6% of S/S, 50% of S/β-thalassemia and 57.1% of S/C patients. These results are contradictory with the hypothesis of splenic regression after the age of five years in children with sickle cell disease 12,14. The persistence of splenomegaly after the age of five years in our patients has already been reported in other African studies supporting the thesis of a possible interaction with malaria 12, 15. Since Malaria is seldom encountered in our country, splenomegaly can be explained by a high level of fetal hemoglobin (HbF) in our patients. The latter could play a role in the persistence of splenomegaly during sickle cell disease 16. According to Serjeant, splenomegaly is usually found in adult S/C patients and has been reported in 50-60% of adult S/C patients 17. In our inquiry, persistence of splenomegaly beyond 5 years of age was statistically more frequent in S/C than in S/S (p^2^ = 0.02). These results are consistent with those found by Diane 18. The persistence of splenomegaly in S/C patients is due to the fact that they have fewer intra splenic falciformations and therefore fewer infarctions that allow them to maintain splenic function until adolescence and adulthood 19. S/β-thalassemia patients have more splenomegaly than S/S patients 18. We found the same results with a statistically significant difference (p1= 0.009). Splenic atrophy was found in 15.1% of patients. It is due to repeated infarctions leading to a progressive decrease in the size of the spleen, resulting in asplenia 14.

Hepatomegaly was noted in 21.21% of patients with an average age of 17.53 ± 5.18 years. The prevalence of hepatomegaly is higher than that found in Manix Ilunga's study which found that 7.76% of his patients had hepatomegaly 20.

Sickle cell crisis is the most common clinical event 21. It is the result of polymerization of HbS in the red blood cell. Thus, as the HbS level increases, the frequency and intensity of attacks increases. This explains the high frequency of Vaso occlusion crisis (VOC) in S/S with the highest HbS levels. In S/C patients' painful episodes occur at about half the frequency as in sickle cell anemia 9. In our work, 30.30% of S/S patients have 1-2 crisis/year and 39.39% have more than three crisis/year. No significant difference was noted between the different phenotypes of SCS. Our results are consistent with those found in Gabon by Moussavou 22.

Acute worsening of anemia is defined as a decrease in baseline Hb of more than 2g/dL. Causes of anemia aggravation in SCS include splenic sequestration, parvovirus B19 infection, bone marrow necrosis, and post-transfusion immunologic events 23. It represents a therapeutic emergency that mainly affects S/S, S/β-thalassemia and S/O_Arab_. S/C patients are the least affected 21. These same facts were found in our work but without a statistically significant difference.

In our study, acute splenic sequestration (ASS) affected six patients at least once (three S/S and three S/β-thalassemia) during their early childhood. The age of onset of the first episode of ASS is quite early (mean age: 4.5± 1.76 years) seeing that 95% of our patients have a Beninese haplotype which is considered severe. This age can be very early, sometimes as early as 5 weeks of age 24. In a Benign study, all cases of ASS occurred within the first year of life 25. In a previous Tunisian study, it was reported that S/C is the least affected 26. Indeed, no cases were noted in our S/C patients.

While in a Senegalese study no cases of ASS were reported in SCS patients, this could be due to the Senegalese haplotype responsible for good tolerance of sickle cell disease 27.

Infections are described as the leading cause of morbidity and mortality in children with sickle cell disease, especially those under 5 years of age 21. In our study, the infectious complications are the third acute complication affecting 44% of patients. In fact, all our patients are properly vaccinated against pneumococcus, meningococcus and haemophilus influenza and they received preventive antibiotic therapy as soon as they were diagnosed.

S/S patients are the most affected (47,22%) because asplenia sets in early stages of the disease. Pulmonary infections predominate the other acute infections such as osteoarticular and urinary infections. Our results are similar to a study conducted in Dakar 28. They differ, from those conducted in Ouagadougou, which reports that infections occur in 90% of cases 8. This high frequency is probably related to the average age of its population, which is much younger than ours and therefore the risk of infection is higher.

Acute Chest Syndrome (ACS) is a serious and frequent complication and life-threatening in SCS, and is the second most common acute complication in sickle cell patients 29. In our work, the frequency was 16.66%, making ACS the fourth most common acute complication. No cases of ACS were noted in S/C patients. According to the literature, ACS can occur in S/C but with a lower frequency than other phenotypes and with the same risk of aggravation 21.

Cerebral vasculopathy mainly concerns S/S and S/β-thalassemia patients 13. S/C patients are not affected, but stroke is possible and is associated with hyperviscosity.

In Tunisia, the frequency of stroke varies: 10.4% as stated by Mellouli 26 and 4% as found by Kouki 30. In our study, we reported a single case of symptomatic stroke in an S/S patient, despite a yearly systematic assessment of the risk of developing a stroke by the request of a transcranial doppler ultrasound (TCD). Mellouli adopted the same strategy as us.

Priapism was noted, in a single homozygous sickle cell boy. This can be explained by the fact that cases of priapism are often not reported spontaneously 22.

Cholelithiasis is a frequent complication of chronic hemolysis in sickle cell patients, the frequency of which increases wit h age. In our study, it is the main chronic complication which was asymptomatic and diagnosed by scheduled yearly abdominal echograhy.

Cholelithiasis was found in 34.84% of patients with a significantly higher frequency in S/S than in S/C (p2=0.014). Hemolysis in S/C patients is more moderate, so that anemia is better tolerated and complications due to hemolysis such as cholelithiasis are less frequent or less severe 9. Parez's study reported a prevalence of cholelithiasis of 15% in S/S patients, and 10% in S/C, with no cases in S/β-thalassemia 31. This low prevalence compared to ours can be explained by the younger age of its patients and by the fact that only 54% of cholelithiasis were diagnosed by systematic echography.

Cardiac complications affect up to 17% of patients, often relative to hyperflow secondary to anemia. These complications are more frequent in homozygous S/S patients 21. 16.66% of our patients had functional systolic murmur with normal or borderline Doppler echocardiography, which is requested every year.

Osteonecrosis affects between 15 and 40% of patients with sickle cell disease and their frequency increases with age 21. They mainly affect the S/S phenotype 26. In our study, this complication affected 6.06% of patients diagnosed by a pelvic X-ray performed systematically every year. Three cases of aseptic osteonecrosis of the femoral heads (two S/S and one S/β-thalassemia) occurred in the second decade of life, and one S/S case of aseptic osteonecrosis of the humeral head occurred at the age of 30 years.

In Tunisia, this complication was found in 4% of patients according to Kouki 31, and 0.95% in Mellouli 26. These differences could be related to genetic, social, and environmental factors. In conformity with the literature, this chronic complication is most common in the S/S and absent in the S/C populations because of near-normal baseline Hb levels and thus good tissue oxygenation 21. In our work, leg ulcer was noted in 3% of the cases. The low frequencies of aseptic osteonecrosis of the femoral head and leg ulcer in our population can be explained by a high HbF level, which would be protective against these complications.

The prevalence of sickle cell nephropathy increases with the age but can also be seen as early as pediatric age. It is diagnosed by microalbuminuria, which is an early and sensitive biological marker of glomerulopathies. The detection of microalbuminuria is carried out systematically in our patients once a year on a 24-hour urine sample. The frequency of nephropathy in S/S patients can be as high as 80% of cases, compared to a much lower prevalence in S/C patients 21. We noted that, sickle cell nephropathy affected only two S/S patients. Microalbuminuria greater than 30mg/24h was noted in ten S/S and seven S/β-thalassemia with a statistically significant difference (p=0.013). Compared to other studies, the frequency of microalbuminuria in ours is relatively low 32.

Sickle cell retinopathy is more common and severe in S/C than in S/S patients.

Dembélé reports an overall prevalence of retinopathy of 8.8%: 12.4% in S/C, 5.2% in S/S and 9.4% in S/β-thalassemia 33. We found only one case of retinopathy in S/S patient (1.51%).

Both in our study and in Dembele's, retinopathy screening is done by a fundus, which is systematically performed once a year.

All clinical data undescribed were noted before taking hydroxyurea by 13 patients. The use of hydroxyurea is not systematic for all patients but only for those who present many Vaso-occlusive crisis that require hospitalization or who have presented ATS, ASS or CVA.

## Conclusion

SCS are characterized by chronic hemolytic anemia with acute and chronic complications. Our study concluded that the clinical data are depending on the phenotype but always dominated by anemia. Severe, acute, and chronic complications affect all phenotypes but moderately in S/C.

It could be explained by the fundamental pathophysiological difference of the S/C disease compared to other SCS phenotypes.

So that, it is important to determine the clinical characteristics of each phenotype of SCS and their evolution in order to be able to provide adequate genetic counselling. The S/C disease being the most moderate form, and therefore can lead an almost normal life. Therefore, medical consultations can be more spaced and the proposal of a prenatal diagnosis for at-risk couples is not essential.

## References

[1] Nicola M, Victoria N, Adekunle A, Kofi AA, Baba I, Biobele B (2016). Proceedings of a Sickle Cell Disease Ontology workshop—Towards thefirst comprehensive ontology for Sickle Cell Disease. Applied & Translational Genomics.

[2] Bibi A, Messaoud T, Beldjord C, Fattoum S (2006). Detection of two rare b-thalassemia alleles found in the Tunisian population: codon 47 (+A) and codon 106/107 (+G). Hemoglobin.

[3] Fattoum S (2006). Les hemoglobinopathies en Tunisie. Revue actualisées des données épidémiologiques et moléculaires. Tunis Med.

[4] Maître B, Mekontso-Dessap A, Habibi A, Bachir D, Parent F, Godeau B (2011). Complications pulmonaires des syndromes drépanocytaires majeurs chez l'adulte. Rev Mal Resp.

[5] Romdhane L, Abdelhak S (2011). Genetic Diseases in the Tunisian Population. Am J Med Genet A.

[6] Ouali F, Siala H, Bibi A, Hadj Fredj S, Dakhlaoui B, Othmani R (2016). Prenatal diagnosis of hemoglobinopathies in Tunisia: 18 years of experience. Int J Tab Hematol.

[7] Mounkaila B, Oumarou Hamido K, Garba M, Abdoulaye Maiga R, AKpona SA, Sanogo I (2015). Hémolyse chronique des sujets drépanocytaires SS et SC en phase stationnaire: étude comparative au centre national de référence de la drépanocytose à Niamey. Rev cames sante.

[8] Ouédraogo-Yugbaré S, Tizndrebeogo J, Koueta F, Sawadogo H, Dao L, Ouédrago P (2014). Syndromes drépanocytaires majeurs chez les enfants de 0 à 15 ans à Ouagadougou: marqueurs génétiques et caractéristiques cliniques. Pan Afr Med J.

[9] Nagel Ronald L, Fabry Mary E, Steinberg Martin H (2003). The paradox of hemoglobin SC disease. Blood Reviews.

[10] Habara AH, Shaikho EM, Steinberg MH (2017). Fetal hemoglobin in sickle cell anemia: The Arab-Indian haplotype and new therapeutic agents. Am J Hematol.

[11] Santos B, Delgadinho M, Ferreira J, Germano I, Miranda A, Arez AP (2020). Co-Inheritance of alpha-thalassemia and sickle cell disease in a cohort of Angolan pediatric patients. Mol Biol Rep.

[12] Thiam L, Dramé A, Zokébé Coly I, Niokhor Diouf F, Seck N, Boiro D (2017). Profils épidemiologiques, cliniques et hématologiques de la drépanocytose homozygote SS en phase inter critique chez l'enfant à Ziguinchor, Sénégal. Pan Afr Med J.

[13] Da Sylva (2015). Profil épidémio-clinique, biologique, thérapeutique et évolutif de la drépanocytose chez l'enfant (expérience de l'unité d'hémato-oncologie du service de pédiatrie du CHU Hassane II. [Thèse].

[14] Beuzard Y, Galactero S (1992). Drépanocytose. Hématologie. Dreyfus et coll Flammarion.

[15] Adekile AD, McKie KM, Adeodu OO, Sulzer AJ, Liu JS, McKie VC (1993). Spleen in sickle cell anemia: comparative studies of Nigerian and US patients. Am J Hematol.

[16] Singer K, Fisher B (1952). Studies on abnormal hemoglobin V. The distribution of types S (sickle cell) hemoglobin and type F (alkali-resistant) hemoglobin within the red cell population in sickle cell anemia. Blood.

[17] Zimmerman SA, War RE (2000). Palpable splenomegaly in children with haemoglobin SC disease: Haematological and clinical manifestations. Clin Tab Haem.

[18] Diagne I, Diagne-Guey, Falla AL, Deme I, Sylla A, Coly JI (2010). Aspects epidemiologiques et evolutifs de la splenomegalie chez les enfants et adolescents porteurs de syndromes drepanocytaires majeurs au Senegal. Arch de Ped.

[19] Lane PA, O'Connell JL, Lear JL, Rogers ZR, Woods GM, Hassell KL (1995). Functional asplenia in hemoglobin SC disease. Blood.

[20] Ilunga Banza M, Panda Mulefu J, Ipani Lire L, Tietie Ben N'dwala Y, Badypwyla Tshiamala I, Kaoma Cabala VP (2019). Pathologies digestives associées à la drépanocytose à Lubumbashi: aspects épidémiologiques et cliniques. Pan Afr Med J.

[21] Habibi A, Arlet JB, Stankovie K, Gellen-Dautremer J, Ribeil JA, Bartolucci P (2015). Recommandation frnçaises de prise en charge de la drépanocytose de l'adulte : actualisation 2015. Rev Med Int.

[22] Moussavou A, Vierin Y, Eloundou-Orima C, Mboussou M, Keita M (2004). Prise en charge de la douleur drépanoctaire selon les critéres de l'Organisation mondiale de la santé. Arch Pediatr.

[23] Arlet JB, Bartoluce P, Habibi A, Ribeil JA, Stankovie K, Lionnet F (2009). L'anémie chez le patient drépanocytaire adulte. Rev Med Int.

[24] Airede Al (1992). Acute splenic sequestration in a five-week-old infant with sickle cell disease. J Pediatr.

[25] Rahimy MC, Gangbo A, Ahouignan G, Adjou R, Deguenon C, Goussanou S (2003). Effect of a comprehensive care programm on disease course in severely ill children with sickle cell anemia in sub-Saharian African setting. Blood.

[26] Mellouli F, Bouzid K, Bejaoui M (2008). Profil évolutif de 105 enfants drépanocytaires tunisiens. Tunis Med.

[27] Diagne I, Ndiaye O, Moreira C, Signate-Sy H, Camara B, Diouf S (2000). Les syndromes drépanocytaires majeurs en pédiatrie à Dakar (Sénégal). Arch Pediatr.

[28] Nacoulma EW, Bonkoungou P, Dembele A, Yé D, Kam L (2006). Les drépanocytoses majeures dans le service de pédiatrie du centre hospitalier universitaire Sourou Sanon de Bobo Dioulasso. Med Afr Noire.

[29] Pincez T, Calamy L, Germont Z, Lemoine A, Lopes AA, Massiot A (2016). Atteintes pulmonaires au cours de la drépanocytose chez l'enfant. Arch Pediatr.

[30] Kouki R, Hichri A, Mokdad A, Bejaoui M (2002). Les syndromes drépanocytaires majeurs : étude de 200 cas. Rev Maghr Pediatr.

[31] Parez N, Quinet B, Batut S, Grimprel E, Larroquet M, Audry G (2001). Lithiase biliaire chez l'enfant drépanocytaire : expérience d'un hopital pédiatrique parisien. Arch Pediatr.

[32] McPherson Yee M, Jabbar SF, Osunkwo I, Clement L, Lane PA, Eckman JR (2011). chronic kidney disease and albuminuria in children with sickle cell disease. Clin J Am Soc Nephrol.

[33] Dembélé AK, Toure BA, Sarro YS, Guindo A, Fané B, Offredo L (2017). Prévalence et facteurs de risque de la rétinopathie drépanocytaire dans un centre de suivi drépanocytaire d'afrique subsaharienne. Rev Med Int.

